# A Whited Sepulchre

**Published:** 1894-04

**Authors:** 


A Whited Sepulchre.—The good old-time preacher said that certain women of the day resembled "a certain green bay tree," notwithstanding its clean and beautiful foliage spread out in the sunlight—and its head carried away up yonder, it was, he said, defective, in that it was "hollow in the butt."

There is a certain medical publication in Cincinnati which may likewise be compared to this same green bay tree, or, more appropriately, to a prostitute all decked out in silks and laces; pretty to look at; but—oh my ! how corrupt !—a very "whited sepulchre." It appears to be a hireling—not a "free," lance, engaged in the unholy cause of defending quackery on the part of once reputable physicians; it attempts to justify the most flagrant violations of the code, and denounces with bitter invectives editors who, very properly, stigmatise such departures as the sale of secret "cures"—as *quackery*. Esau was not so unreasonable, we apprehend, as to want to hold on to his birth-right while luxuriating in his soup, and there was none to demand it for him. Renegades to medicine should accept, with the price, the consequences; when one turns quack he should not be surprised to find himself kicked out of the profession, and no plea of former respectability—not even of seventeen years' record as a college professor, should entitle him to have his cake, and eat it too. But a new species of journalism has arisen in Cincinnati, it seems—one of the Ishmael variety; its hand is against all reputable medicine. The press should give it its deserts.

